# Radioprotective Effect of Whey Hydrolysate Peptides against γ-Radiation-Induced Oxidative Stress in BALB/c Mice

**DOI:** 10.3390/nu13030816

**Published:** 2021-03-02

**Authors:** Xin-Ran Liu, Na Zhu, Yun-Tao Hao, Xiao-Chen Yu, Zhen Li, Rui-Xue Mao, Rui Liu, Jia-Wei Kang, Jia-Ni Hu, Yong Li

**Affiliations:** Department of Nutrition and Food Hygiene, School of Public Health, Peking University, Beijing 100191, China; 1811110178@bjmu.edu.cn (X.-R.L.); 1911110164@bjmu.edu.cn (N.Z.); haoyuntaolly@bjmu.edu.cn (Y.-T.H.); 1410306228@pku.edu.cn (X.-C.Y.); 1410306201@pku.edu.cn (Z.L.); maoruixue@bjmu.edu.cn (R.-X.M.); 2016391040@bjmu.edu.cn (R.L.); kjiawei@yeah.net (J.-W.K.); 1811210226@bjmu.edu.cn (J.-N.H.)

**Keywords:** whey hydrolysate peptides, radioprotective effect, γ-radiation-induced, oxidative stress

## Abstract

Radiation therapy is widely used in the treatment of tumor diseases, but it can also cause serious damage to the body, so it is necessary to find effective nutritional supplements. The main purpose of this study is to evaluate the protective effect of whey hydrolysate peptides (WHPs) against ^60^Coγ radiation damage in mice and explore the mechanism. BALB/c mice were given WHPs by oral gavage administration for 14 days. Then, some mice underwent a 30-day survival test after 8 Gy radiation, and other mice received 3.5 Gy radiation to analyze the changes in body weight, hematology and bone marrow DNA after three and 14 days. In addition, through further analysis of the level of oxidative stress and intestinal barrier function, the possible mechanism of the radioprotective effect of WHPs was explored. The study found WHPs can prolong survival time, restore body weight, and increase the number of peripheral blood white blood cells and bone marrow DNA content in irradiated mice. In addition, WHPs can significantly improve the antioxidant capacity, inhibit pro-inflammatory cytokines and protect the intestinal barrier. These results indicate that WHPs have a certain radioprotective effect in mice, and the main mechanism is related to reducing oxidative damage.

## 1. Introduction

At present, nuclear energy and radiation play a crucial role in the treatment of malignant tumors. Cancer is the second leading cause of death in the world, with an estimated 10 million deaths in 2018, accounting for one sixth of all deaths [1]. In the comprehensive treatment of cancer, 70–80% of patients need radiotherapy and chemotherapy, and this proportion is increasing year by year. A commonly used radiotherapy source is gamma beams from a radioactive Cobalt-60. Therapeutic doses of radiation during routine procedures range from 20 to 60 Gy [2,3]. While we profit from the great benefits of ionizing radiation, we also inevitably suffer from the direct or indirect radiation damage or radiation pollution to the environment and the human body. Radiotherapy and chemotherapy not only kill cancer cells, but also damage normal cells and tissues of the organism. They cause many side effects, such as decreased immunity, massive reduction of white blood cells, gastrointestinal dysfunction and production of reactive oxygen species (ROS) in patients. Acute radiation syndrome (ARS) is a complex interaction of main symptoms of cancer patients after radiotherapy, including fatigue, loss of appetite, nausea, vomiting, diarrhea, fewer white blood cells and decreased immune function. Data show that acute radiation injury can be caused at the radiation dose of 1 Gy, and the radiation injury is aggravated with the increase of the absorbed doses [4]. 

Scavenging free radicals is the primary mechanism of radiation protection [5]. Many natural antioxidants can not only scavenge the free radicals produced by ionizing radiation, but also minimize the complications of oxidative stress. Thus, it is of great significance to recognize new radioprotective molecules based on this mechanism. Peptide, with relatively low molecular weight, is a hydrolytic form of protein. Previous studies have shown that bioactive peptides have health-promoting benefits and biological activities, such as antioxidant, antibacterial, immunomodulatory and anticancer effects [6]. In addition, the European Society of Parenteral and Enteral Nutrition advocates that cancer patients with nutritional risk or malnutrition need oral nutritional supplements (ONS) as nutritional intervention when receiving radiotherapy. Meanwhile, nutritional support should provide protein with a target supply of 1.2–2 g/kg per day to minimize weight loss, reduce the negative impact of radiotherapy and maintain quality of life [7]. Exhilaratingly, peptides have better functional properties than the parent proteins and are thus used in the food industry for various purposes [8]. Bioactive peptides have been paid great attention by the medical and health care industry because of their rich resources and fewer side effects. Therefore, protein-derived bioactive substances can not only meet the protein intake of patients, but also produce more bioactive effects to repair radiation damage.

Whey protein is a kind of high-quality protein. It is mainly composed of α-lactalbumin and β-lactoglobulin, accounting for 70–80% of the total protein. Other components are bovine serum albumin, lactoferrin, lactoperoxidase, immunoglobulin, growth factor and a multitude of bioactive peptides. Whey hydrolysate peptides (WHPs) can be obtained by hydrolyzing the intact whey protein into smaller bioactive fragments, which can be easy to digest and absorb, especially for cancer radiotherapy patients with diminished digestive function. Many studies have confirmed that whey protein-based peptides have the ability of anti-oxidation, anti-inflammation and immune regulation, but there has been no previous evidence of their role and mechanism on radioprotection [9,10,11]. This study sought to assess the radioprotective effect of WHPs in irradiated mice and explored possible mechanisms for the role of WHPs. Specifically, our research focuses on analyzing the radioprotective effect of WHPs on mice and their mechanism under a sublethal dose of radiation. In addition, we use a lethal radiation dose that can induce 100% mortality to observe whether WHPs can prolong the survival time of mice in this condition.

## 2. Materials and Methods

### 2.1. Materials and Reagents

WHPs (Hilmar product 8350) are a mixture of small bioactive peptides hydrolyzed from whey protein purchased from Tianjin Milkyway Import & Export Corp. (Tianjin, China). The degree of hydrolysis was 12.5%, and the content of protein of the dry weight was 82%. The amino acid composition can be seen in Table 1. The content with a relative molecular weight less than 5000 Dalton was 67.2%, and the content with a relative molecular weight less than 1000 Dalton was 40.5%.

Whey protein concentrate (Hilmar product 8200), used as an intact protein contrast, had approximately the same total protein content as WHPs and very few peptides. It also was purchased from Tianjin Milkyway Import & Export Corp.

Nutritional assessment and estimated total protein intake in each group can be found in Supplementary File S1. The content of total protein intake was obtained by determining the nitrogen content of the original protein in AIN-93G diet and the amount of supplementary peptide or protein, depending on the different group setting, and multiplying the value by 6.25. No significant difference was found in protein intake among the groups, and the actual maximum intake of total protein after protein supplementation did not exceed the blank control, which was a suitable and acceptable nutritional state.

### 2.2. Animals and Treatments

**Figure 1:**
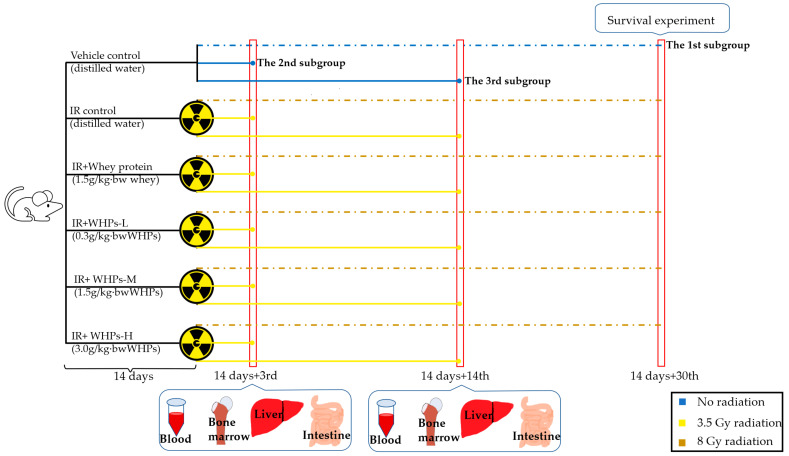
Study process diagram. IR, irradiated mice; IR+WHPs-L, irradiated mice+low-dose whey hydrolysate peptides; IR+WHPs-M, irradiated mice+medium-dose whey hydrolysate peptides; IR+WHPs-H, irradiated mice+high-dose whey hydrolysate peptides.

The study process is shown in Figure 1. A total of 216 female BALB/c mice weighing 18 to 20 g were purchased from the Animal Service of Health Science Center, Peking University. All mice were given free access to food (AIN-93G diet) and distilled water and housed at a temperature of 24 ± 2 °C, a relative humidity of 50 ± 10% and a 12 h:12 h light-dark cycle. Principles of Laboratory Animal Care (NIH publication No. 85-23, revised 1985) were followed, and all procedures were conducted according to the guidelines established by Peking University Animal Research Committee. The laboratory animal production license number is SCXK (Jing) 2016-0010, and laboratory animal use license number is SYXK (Jing) 2016-0041. 

After a one-week period of adaptation, all mice were randomized into 6 groups (*n* = 36): vehicle control group (not exposed to radiation), IR control group (irradiated mice model without any nutritional intervention), IR+whey protein group (irradiated mice were given intact whey protein intervention) and three IR+WHPs groups (IR+WHPs-L, IR+WHPs-M, IR+WHPs-H represent low, medium and high doses of WHP intervention in irradiated mice, respectively). In the next 14 days, mice in the vehicle control group and IR control group were orally administered by gavage with distilled water. The IR+whey protein group was orally administered by gavage with 1.5 g/kg body weight whey protein concentrate aqueous solution, while the three IR+WHPs groups ingested 0.3, 1.5, and 3.0 g/kg body weight WHP aqueous solution in the same way.

After 14 days of daily administration, each group was divided randomly into 3 subgroups (*n* = 12) for subsequent different treatment. The mice of the first subgroup, except for the vehicle control group, received a lethal dose of 8 Gy ^60^Co-γ radiation (1 Gy/min) for the survival experiment [12]. In the other two subgroups, except for the vehicle control group, mice were exposed to 3.5 Gy radiation (1 Gy/min). On the 3rd day after irradiation, all mice in the second subgroup were sacrificed for the subsequent tests. Mice in the third subgroup were sacrificed for the subsequent tests on the 14th day after irradiation. During the radiation and observation period, each group of mice still maintained the corresponding intervention. 

### 2.3. Irradiation Process

Radiation was performed by ^60^Co-γ radiation facility (Reviss Services Ltd., Abingdon, UK) in the Radiation Center (Peking University, Beijing, China). Mice were placed in separate plexiglass chambers (3 cm × 3 cm × 11 cm) and given ^60^Co-γ total body irradiation (TBI).

### 2.4. Experimental Assays

#### 2.4.1. Survival Experiment

In the first subgroup, the survival time of mice among each group was recorded after being radiated with 8 Gy ^60^Co-γ radiation.

#### 2.4.2. General Status, Body Weight and Organ Index

In the second and third subgroups, the general status of mice was observed after being radiated with 3.5 Gy ^60^Co-γ radiation. The body weight of mice was recorded at the beginning of the intervention and at the end point. Mice of the second subgroup were sacrificed 3 days after radiation, while mice of the third subgroup were sacrificed 14 days after. The spleen, thymus and liver were removed and weighed immediately after the mice were sacrificed. An organ index was calculated by their weight relative to the final body weight (thymus weight/body weight; spleen weight/body weight).

#### 2.4.3. Peripheral White Blood Cell Count and Bone Marrow DNA Content

On the 3rd and 14th day after radiation, the peripheral blood of mice was collected via the tail vein and added to 1% hydrochloric acid solution. The white blood cells (WBCs) were counted using an automated hematology analyzer (Beckman Coulter Inc., Brea, CA, USA). 

The bone marrow of mice was collected immediately after the mice were sacrificed. The bone marrow cells were flushed out with Hanks solution and collected for DNA damage analysis. The DNA concentration was measured by a FlexStation2 fluorescence microplate reader (Molecular Devices, Sunnyvale, CA, USA).

#### 2.4.4. Analysis of Superoxide Dismutase (SOD), Glutathione Peroxidase (GSH-PX) and Malondialdehyde (MDA) Levels

Mice were sacrificed on the 3rd day and 14th day after radiation. Blood was obtained via the orbital sinus and then centrifuged at (3000 rpm, 10 min) for preparing the serum. The livers of the mice were collected immediately after the mice were sacrificed for preparing tissue homogenates. SOD, GSH-PX and MDA levels in serum and liver were detected using corresponding ELISA Kits (Dogesce, Beijing, China) and automatic multi-function microplate reader multiskan MK3 (Thermo, Waltham, MA, USA). 

#### 2.4.5. Analysis of Tumor Necrosis Factor Alpha (TNF-α) and Interleukin-6 (IL-6) Levels

The preparation of the mouse serum is as described in Section 2.4.4. TNF-α and IL-6 concentrations in serum were detected using corresponding ELISA Kits (Dogesce, China) and automatic multi-function microplate reader multiskan MK3 (Thermo, USA). 

#### 2.4.6. Histopathology of Mouse Small Intestine

Immediately after sacrificing, the intestines of mice were fixed with 10% (*v*/*v*) formaldehyde. Fixed tissues were taken out and soaked in distilled water overnight. Then, they were dehydrated with 70% and 80% ethanol for 2 h, immersed in xylene, and embedded in melted paraffin for 16 h. After vacuum infiltration at 60 °C, embedded wax blocks were cut into 5 μm sections with Thermo Scientific Microm HM325 rotary microtome (Thermo, USA). Tissue sections were dewaxed with xylene twice, placed in absolute ethanol, 95% ethanol, 80% ethanol and 70% ethanol for 1–2 min and then washed with distilled water for 3 min. After being stained with hematoxylin solution for 5 min and differentiated with 1% acid ethanol, sections were washed in running water for at least 15 min until the nucleus turned blue. Then, sections were stained with eosin solution and dehydrated twice with 95% ethanol and absolute ethanol. Finally, the sections were immersed in phenol xylene and xylene in sequence, and sealed with neutral gum.

After paraffin embedding, sectioning, and H&E staining, images for histological slides were taken at 200× magnification by Nikon Eclipse E400 biological microscope (Nikon, Tokyo, Japan) with Nikon Digital Camera DXM1200F (Nikon, Japan). Villus length and crypt depth of the intestine were analyzed by Image J 1.52v (Wayne Rasband National Institutes of Health, Bethesda, MD, USA). 

#### 2.4.7. Expression of Occludin and Zona Occludens 1(ZO-1) in Intestine

Expression of occludin and ZO-1 in the intestine were detected by immunohistochemistry, using rabbit polyclonal antibody against occluding (BIOSS, Beijing, China, 1:200 dilution) and ZO-1 (BIOSS, China,1:500 dilution). Slides were evaluated by Image-Pro Plus version 6.0 (Media Cybernetics Inc., Bethesda, MD, USA), calculating the mean of optical density. 

#### 2.4.8. Analysis of Serum D-Lactic Acid, Diamine Oxidase (DAO) and Lipopolysaccharide (LPS) Levels

The preparation of the mouse serum is as described in Section 2.4.4 above. D-lactic acid, DAO and LPS concentrations in serum were detected using the corresponding ELISA kits (Dogesce, China) and automatic multi-function microplate reader multiskan MK3 (Thermo, USA). 

### 2.5. Statistical Analysis

The experimental observations were expressed as mean ± standard deviation ($\bar{X}$ ± s) or median (Q25, Q75). Data were analyzed by the one-way analysis of variance (ANOVA) method followed by Tukey’s post-test or Dunnett’s T3 test. Lifetime data were estimated with the Kaplan–Meier analysis for survival curves, and differences among groups were compared by the Wilcoxon rank sum test. All statistical analyses were performed using IBM SPSS 23 statistics software (IBM Corp, Armonk, NY, USA), with a significance value of *p* < 0.05.

## 3. Results

### 3.1. WHPs Prolong the Survival Time of Irradiated Mice

**Figure 2:**
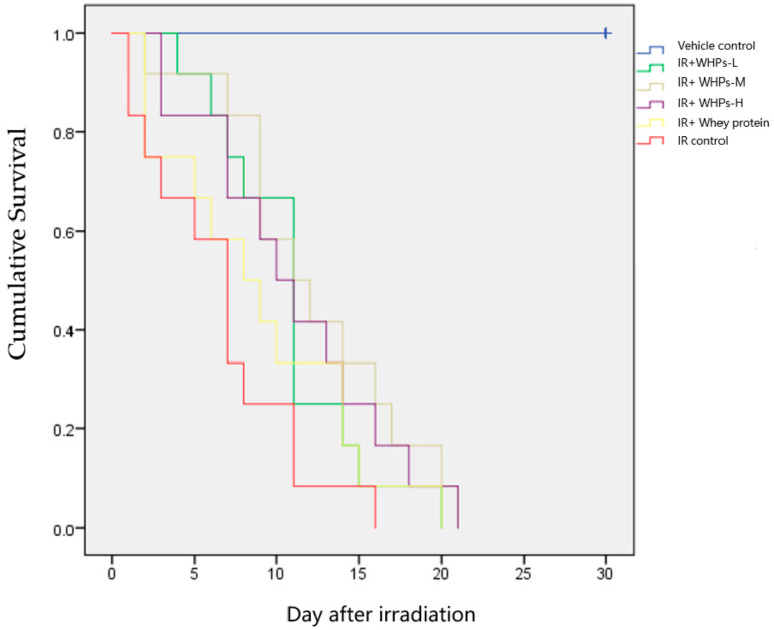
Kaplan–Meier survival curves of mice in each group. IR, irradiated mice; IR+WHPs-L, irradiated mice+low-dose whey hydrolysate peptides; IR+WHPs-M, irradiated mice+medium-dose whey hydrolysate peptides; IR+WHPs-H, irradiated mice+high-dose whey hydrolysate peptides.

After 8 Gy radiation exposure, the irradiated mice gradually died the next day; the survival curves are shown in Figure 2. As shown in Table 2, compared with the vehicle control group, the survival time of irradiated mice in other groups was significantly shortened (*p* < 0.05). Compared with the IR control group, the survival time of mice in the medium-dose WHP group (IR+WHPs-M) was significantly prolonged (*p* < 0.05).

### 3.2. WHPs Restore Body Weight in Irradiated Mice

**Figure 3:**
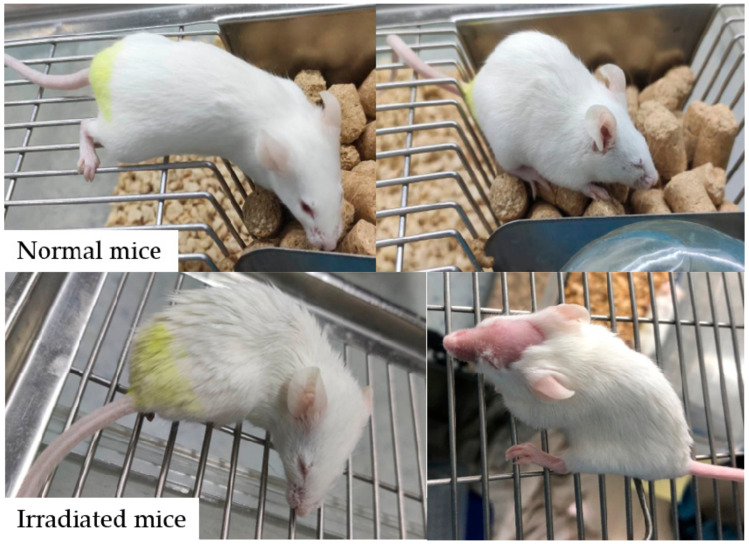
General status of normal mice and irradiated mice.

Irradiated mice showed disease symptoms that included anorexia, listlessness, decreased drinking of water, emaciation, tarnishing of hair, diarrhea and drowsiness (Figure 3). One mouse in the IR control group was found to be shedding hair on the seventh day after 8 Gy radiation, and one mouse in the IR+WHP-L group was found to be shedding hair on the 12th day after 8 Gy radiation. However, there was no significant difference in the appearance of mice among the intervention groups.

On the third day after radiation, there was no significant difference in body weight among the groups. On the 14th day after radiation, body weight of the IR control group was significantly lower than that of the vehicle control group, while the IR+WHPs-M group had diminished weight loss (seen in Table 3).

As seen in Table 4 and Table 5, on the third day after irradiation, the spleen and thymus indexes of the irradiated mice were significantly lower than those of the vehicle control group, but there was no difference among the intervention groups. By the 14th day after radiation, the organ indexes had recovered on their own. The spleen index in the whey protein intervention group was higher than that in the vehicle control group, suggesting that there might be splenomegaly (Table 5). Compared with the IR control group, the low-dose WHP intervention (IR+WHPs-L) significantly reduced the liver index, suggesting that the intervention of WHPs may have a protective effect on the liver.

### 3.3. WHPs Accelerate the Recovery of Peripheral WBC in Irradiated Mice

As shown in Table 6, on the third day after radiation, compared with the vehicle control group, the number of WBCs in peripheral blood of irradiated mice in each group was significantly reduced (*p* < 0.05), but there was no significant difference among the intervention groups. On the 14th day after radiation, although the WBCs of the mice recovered to a certain extent, the number of WBCs in the peripheral blood of the irradiated mice was still lower than normal. Compared with the IR control group, the number of peripheral blood WBCs in the high-dose WHP group was significantly increased (*p* < 0.05).

### 3.4. WHPs Promote the Recovery of Bone Marrow DNA Damage in Irradiated Mice

**Figure 4:**
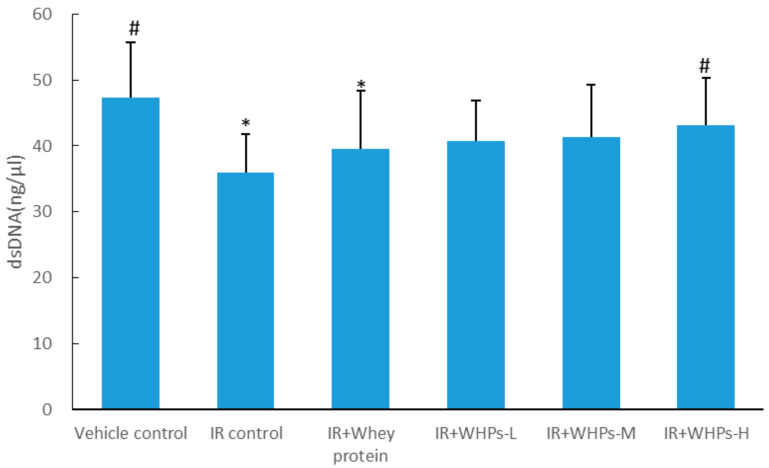
Effect of WHPs on DNA content in bone marrow at day three after radiation. * The difference was statistically significant (*p* < 0.05) compared with the vehicle control group; # the difference was statistically significant (*p* < 0.05) compared with the IR control group. IR, irradiated mice; IR+WHPs-L, irradiated mice+low-dose whey hydrolysate peptides; IR+WHPs-M, irradiated mice+medium-dose whey hydrolysate peptides; IR+WHPs-H, irradiated mice+high-dose whey hydrolysate peptides.

As shown in Figure 4, on the third day after radiation, the bone marrow DNA content of mice in the IR control group and whey protein control group (*p* < 0.05) was significantly lower than that of vehicle control group mice. Compared with the IR control group, the bone marrow DNA content of mice in the high-dose WHP group was significantly increased (*p* < 0.05).

### 3.5. WHPs Improve the Antioxidant Capacity of Irradiated Mice

According to Table 7 and Table 8, both on the third and 14th day after radiation, the activities of SOD and GSH-PX in serum and liver of the IR control group were significantly decreased, and MDA content in liver and serum were significantly increased (*p* < 0.05). 

Whether in the serum or in the liver, SOD and GSH-PX activity of mice in each WHP group increased significantly to varying degrees, accompanied by a significant decrease in serum and liver MDA content. Moreover, the antioxidant effect on the 14th day is directly in line with the findings on the third day. Therefore, WHPs show good antioxidant activity and can repair the oxidative damage caused by radiation to a great extent.

### 3.6. WHPs Regulate the Level of Inflammatory Cytokines in Irradiated Mice

**Figure 5:**
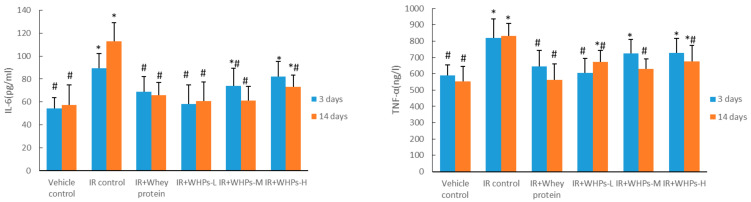
Effect of WHPs on inflammatory cytokine levels in serum. * The difference was statistically significant (*p* < 0.05) compared with the vehicle control group; # the difference was statistically significant (*p* < 0.05) compared with the IR control group. IR, irradiated mice; IR+WHPs-L, irradiated mice+low-dose whey hydrolysate peptides; IR+WHPs-M, irradiated mice+medium-dose whey hydrolysate peptides; IR+WHPs-H, irradiated mice+high-dose whey hydrolysate peptides.

As shown in Figure 5, the serum IL-6 level of the IR control group mice and whey protein group mice was significantly higher than that of the vehicle control group (*p* < 0.05). Compared with the IR control group, the serum IL-6 level of IR+WHPs-L, IR+WHPs-M and whey protein group mice was significantly decreased on the third day after radiation. By the 14th day, the serum IL-6 level in all intervention group mice was significantly decreased (*p* < 0.05).

Similarly, radiation significantly increased TNF-α content of the IR control group mice. Compared with the IR control group, the serum TNF–α level of the low-dose WHP and IR+whey protein group mice was significantly decreased on the third day after radiation, and was significantly decreased for all groups on the 14th day after radiation (*p* < 0.05).

### 3.7. WHPs Protect Intestinal Morphology and Structure in Irradiated Mice

**Figure 6:**
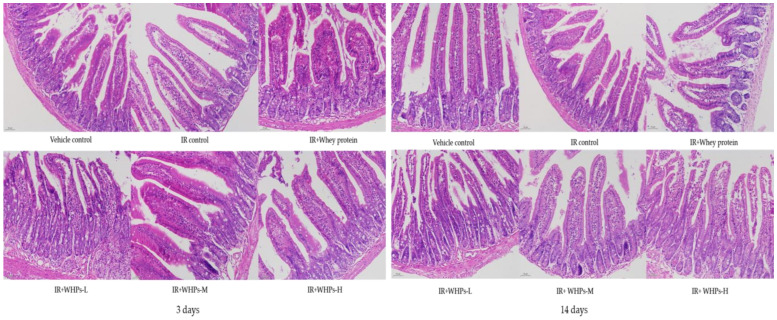
Effect of WHPs on intestinal morphology in irradiated mice (HE staining of sections, magnification: 200×). IR, irradiated mice; IR+WHPs-L, irradiated mice+low-dose whey hydrolysate peptides; IR+WHPs-M, irradiated mice+medium-dose whey hydrolysate peptides; IR+WHPs-H, irradiated mice+high-dose whey hydrolysate peptides.

As shown in Figure 6, the villus of the small intestine of the vehicle control group was arranged tightly and regularly. The intestinal glands were complete. In the IR control group, the villus was sparse, irregular, atrophic and broken, with fewer residual intestinal glands. Whey protein and WHPs improved the intestinal morphology and structure of mice to certain degrees. 

As shown in Table 9, on the third day after radiation, compared with the vehicle control group, the length of the small intestinal villus in the IR control group was significantly reduced. Compared with the IR control group, the length of the small intestinal villus in the high-dose WHP group was significantly increased, and the depth of the crypt in the high-dose WHP group was significantly decreased (*p* < 0.05). On the 14th day after irradiation, compared with the vehicle control group, the crypt depth in the IR control group was significantly increased, but there was no statistical difference between the other groups and the IR control group. Although WHPs have a milder effect on villus and crypts overall, they still show an improvement trend. It is suggested that the intervention of WHPs is helpful to the recovery of intestinal morphology.

### 3.8. WHPs Increase Intestinal Tight Junction Protein Expression in the Acute Phase of Radiation Injury

**Figure 7:**
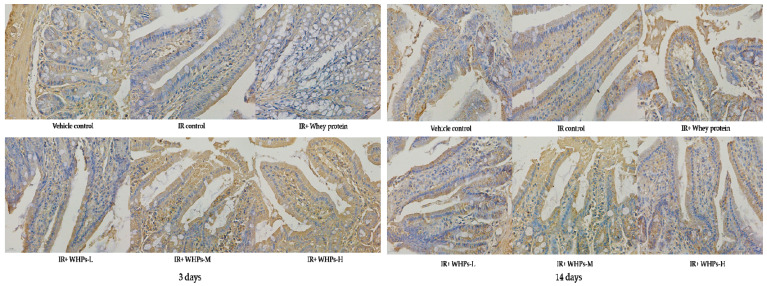
Effect of WHPs on the expression of occludin proteins in mice (400×). IR, irradiated mice; IR+WHPs-L, irradiated mice+low-dose whey hydrolysate peptides; IR+WHPs-M, irradiated mice+medium-dose whey hydrolysate peptides; IR+WHPs-H, irradiated mice+high-dose whey hydrolysate peptides.

**Figure 8:**
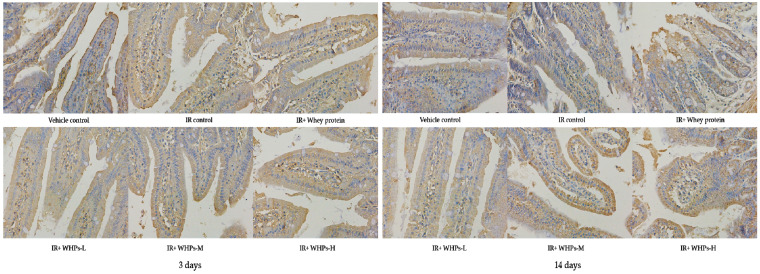
Effect of WHPs on the expression of ZO-1 proteins in mice (400×). IR, irradiated mice; IR+WHPs-L, irradiated mice+low-dose whey hydrolysate peptides; IR+WHPs-M, irradiated mice+medium-dose whey hydrolysate peptides; IR+WHPs-H, irradiated mice+high-dose whey hydrolysate peptides.

On the third day after irradiation, the expression of occludin and ZO-1 in the IR control group was significantly lower than that in the vehicle control group (*p* < 0.05). Compared with IR control group, ZO-1 expression in the high-dose WHP group was significantly increased (*p* < 0.05) (see Figure 7, Figure 8, Table 10). After 14 days of radiation, there was no significant difference in expression of occludin and ZO-1 proteins among the groups, suggesting there was a certain degree of self-recovery after injury.

### 3.9. Effect of WHPs on Permeability in Irradiated Mice Intestinal Permeability

As shown in Table 11 and Table 12, there was no significant difference of the serum D-Lactate, LPS and DAO content among the groups. It is suggested that this dose of radiation did not cause a serious increase in intestinal permeability, so we could not determine the intervention effect of WHPs.

## 4. Discussion

Despite the wide use of whey protein hydrolysate as infant formulas to promote health, the therapeutic potential of WHPs as a radioprotective agent is poorly understood [13]. Damage from radiation on mice is reflected in all aspects of body function, but the significant shortening of survival is the most obvious. The 30-day survival experiment after whole body radiation is the most commonly used test [14]. In this study, we exposed mice to 8 Gy of radiation to induce 100% mortality in mice. It is known that such a lethal dose of radiation can induce hematopoietic syndrome, accompanied by systemic and organ-specific inflammatory reactions, leading to multiple organ failure and ultimately death [15]. The present study is along similar lines. The result of this study showed that all mice in the IR control group died within 16 days after 8 Gy radiation exposure, while the average survival time of the mice in the WHPs group was extended by 3–4 days. These findings suggest that WHPs significantly improve the survival of irradiated mice.

Since the radiation dose absorbed by people under planned exposure is unlikely to be fatal, more attention is needed to investigate the impact of sub-lethal radiation doses. Therefore, our research focuses more on the radioprotective effect of sublethal doses. On the basis of the previous immunosuppressive studies of our research team, combined with the results of published articles on radiation damage in mice, we appropriately reduced the radiation dose to 3.5 Gy [16]. The results of this experiment showed that this dose of radiation caused acute damage to the immune organs of mice, and no mice died during the observation period. It is appropriate to study the moderate degree of radiation damage of sublethal doses and the repairing effect of the peptides. 

An intuitive manifestation of sublethal radiation is weight loss. Radiation caused damage to the gastrointestinal mucosa, resulting in absorption disorder and ultimately leading to restricted weight gain of irradiated mice. The body weight results on the 14th day after radiation showed that radiation significantly reduced the body weight of mice, while the weight of mice in the intervention group of the IR+WHPs-M recovered significantly. WHPs, as nutrients in the form of protein hydrolysis, can compensate for weight loss. In addition, peptides may repair the intestinal damage and thus restore the body weight of irradiated mice. 

Using intact whey protein as a control for our experiments with WHPs, we can exclude the possibility of obtaining false positive results that may be caused by an increased protein intake. The survival time and body weight of the IR+WHPs-M mice were greater than those of the IR+whey protein group mice, indicating that peptides play a better role than intact protein in the nutritional support of irradiated mice. In addition, we found that the relative weight of the spleen in IR+whey group mice increased 14 days after radiation, suggesting that whey protein supplementation may cause spleen inflammation, but WHPs did not [17]. Interestingly, the effects of WHPs on survival time and body weight did not follow a dose-response relationship. We speculate that this may be due to the relatively high viscosity of the high-dose WHP solution. In this study, the daily intake of WHPs was taken by one-time intragastrical administration, which may not be conducive to its digestion and absorption process. In future studies, it can be improved to two intragastrical administrations a day.

The earliest changes caused by radiation occurred in the blood system and hematopoietic system. The damage of hematopoietic parenchymal cells and the destruction of the hematopoietic microenvironment leads to hematopoietic failure, which can be reflected in changes in peripheral blood components. White blood cells have a short lifespan. Under radiation exposure, damaged hematopoietic stem progenitor cells reduce blood cell production, and the number of white blood cells in peripheral blood drops sharply [18]. Bone marrow is a particularly sensitive tissue to radiation, as well as an important organ of hematopoiesis and immunity. DNA is an important class of biological macromolecules in the body. A large number of free radicals are generated during the radiation process, which then attack the DNA molecular structure of the body’s cells. It can destroy the base and DNA strand, interfere with DNA replication and significantly reduce the double-stranded DNA (dsDNA) content of bone marrow cells [19]. Therefore, bone marrow dsDNA is an important indicator for evaluating the degree of bone marrow damage and also a target for radiation protection. In this study, the dsDNA content of bone marrow cells was increased by different doses of WHPs. The dsDNA content of bone marrow cells in the IR+WHPs-H group mice was significantly higher than that of the IR control group, and its content had returned to the level of the vehicle control group. Our data suggested that WHPs could repair damage of the hematopoietic system through increasing the dsDNA content of bone marrow and accelerating the recovery of peripheral WBCs. The mechanism may be related to scavenging free radicals and repairing DNA lesions.

According to reports, most of the DNA damage caused by ionizing radiation is induced by free radicals [20]. Free radicals are molecules responsible for aging and tissue damage. Excessive free radicals produced by radiation can cause tissue damage, leading to hematopoietic suppression, immunosuppression and intestinal barrier dysfunction. The radiation damage mainly comes from the ROS, a kind of oxygen-related free radical produced by ionization of water in the body tissues [21,22]. MDA is a lipid peroxide produced by free radicals acting on polyunsaturated fatty acids in biological membranes. Its content in serum and tissue shows the degree of lipid peroxidation, which can be used as an indicator of the content of free radicals. SOD and GSH-PX are important antioxidant enzymes in the antioxidant defense system, minimizing the interference caused by ROS. SOD can convert O2¯ into H_2_O_2_ through disproportionation reaction, and then convert into H_2_O and O_2_ by GSH-PX and catalase (CAT) [23]. The antioxidant effect of bioactive peptides has been widely studied, and WHPs also showed strong antioxidant activity in this study. WHP intervention increased the activities of SOD and GSH-PX in the serum and liver and significantly decreased the content of MDA in the serum and liver. The volume of the liver increases in a state of damage such as peroxidation. The liver index in the IR+WHPs-L group (see Table 5) was significantly lower than that of the IR control group; thus, we speculate that WHPs can protect the liver, and the mechanism is related to the antioxidant response.

ROS can promote the expression of pro-inflammatory TNF-α and IL-6 through activating the transcription factor nuclear factor-kappa B (NF-κB), and become the initiating factor of early inflammation [24]. At the same time, the increase of inflammatory mediators can further aggravate oxidative stress [25]. Therefore, there is a close connection between inflammation and oxidative stress, forming a vicious circle in radiation damage. Results indicated that WHPs effectively reduced the level of pro-inflammatory cytokines and alleviated inflammatory response in irradiated mice. Oxidative stress caused by excess ROS accelerates telomere shortening and leads to a gradual decline of cellular functions and eventual death [26]. Elevated pro-inflammatory markers (such as IL-6) are also a strong risk factor for geriatric conditions, including premature death [27]. Therefore, WHPs showed an effective regulation of oxidative stress and inflammation, which explained the results of prolonged survival time found in the study.

The intestines are also an important immune organ of the human body. There are four barriers, including the mechanical barrier, chemical barrier, biological barrier and immune barrier, in the intestines to prevent the invasion of pathogens. Among them, the mechanical barrier is the foundation of the entire intestinal structure. It is composed of intestinal mucosal epithelial cells and tight connections between cells. The intestinal epithelium is covered by a single layer of columnar epithelial cells and can be divided into crypts and villus. The crypts invert into the lower mesenchyme, with the villus extending into the intestinal cavity. Tight junction proteins are mainly composed of occludin and ZO families. The direct damage to the crypt epithelium and the subsequent destruction of the villus epithelium are the main reasons for the abnormal morphology of the intestinal mucosa caused by radiation. At the same time, radiation can interfere with mucus and enzymes secreted by the crypt tissues, which constitute a chemical barrier. Therefore, radiation damage to villus and crypts needs to be taken seriously. Our current study found that 3.5 Gy of radiation caused irregularity, sparsity, atrophy, and even breakage of the small intestinal villus of mice. Compared with normal mice, the length of the villus was significantly reduced, and the depth of crypts was significantly increased. However, in some groups treated with WHPs, the villus length of mice increased and the depth of crypts decreased, indicating that the intervention of WHPs helped to restore the structure of the small intestine to varying degrees. At the same time, we found that the expression of ZO-1 in mice in the IR+WHPs-H group was significantly higher than that of the IR control group after three days of radiation, indicating that this dose of WHPs intervention has a certain effect on up-regulated expression of the tight junction protein. On the 14th day after radiation, the effect of WHP intervention was not reflected in the protein expression because of the self-repair capability of the intestine.

The destruction of the intestinal barrier will lead to an increase in intestinal permeability. The content of substances such as DAO and D-lactic acid, which are originally very low in the blood, will increase significantly. A large number of bacteria and endotoxins translocate into the blood, causing endotoxemia and lipopolysaccharide in the blood. The content of toxins’ LPS increased significantly, and led to excessive release of inflammatory mediators and cytokines, further aggravating the intestinal mucosal barrier damage [28]. The levels of serum D-Lactate, DAO and LPS can reflect the intestinal permeability and intestinal damage. Our study did not find any significant difference in content among groups, indicating that the radiation damage at a dose of 3.5 Gy has not yet affected the intestinal permeability. Therefore, we believe that future studies need to use higher radiation doses to find changes in intestinal permeability.

Based on the results of this study, we believe that the mechanism by which WHPs play a role in radioprotection is to increase their antioxidant activity and anti-inflammatory ability. We further speculate that this is because WHPs are rich in small-molecule bioactive peptides. Analysis of the amino acid profile of WHPs reveals that they are rich in precursor substances that synthesize the well-known antioxidant glutathione [29]. Glutathione (γ-glutamyl-cysteinyl-glycine) is a small, low-molecular weight, water-soluble thiol-tripeptide formed by three amino acids (glutamate, cysteine and glycine). Among them, cysteine is the restrictive component for the synthesis of glutathione, and WHPs contain more cysteine and glutamic acid compared to other proteins sources. Many studies have shown also that whey protein can significantly increase glutathione levels, especially in immune deficiency states [30]. In addition, the absorption and utilization process of peptides as protein hydrolysates in the gastrointestinal tract has more kinetic advantages than intact proteins and may maximize any physiological effects [31].

Although WHPs may not have the efficacy of synthetic drugs, they usually have no side effects and therefore represent a natural health-promoting supplement, currently demanded by consumers worldwide [32]. In addition, since WHPs are a mixture of peptides, when WHPs are ingested, there may be significant potential for synergistic effects of multiple peptides. However, for more in-depth research in the future, analyzing the bioactivity sequence of target peptides by LC-MS/MS analysis, exploring amino acid kinetics and examining more thorough damage effects, such as on bone marrow and the intestines, are the next steps to be explored. Our research examined the amount of DNA extracted from bone marrow, using the ultraviolet light absorption method (A 260). This method can reflect the amount of double-stranded DNA contained in bone marrow cells. This test is simple and quick, and it has been used previously in published research. However, the results from this test may be contaminated by some single-stranded DNA. In future studies, the γ-H2AX staining method should be used to evaluate DNA damage.

## 5. Conclusions

The current results are sufficient to point out that WHP intervention has a certain protective effect in irradiated mice. Specifically, WHPs can prolong the survival time after radiation, restore the body weight, increase the number of peripheral WBC and bone marrow dsDNA content, improve the antioxidant capacity, inhibit pro-inflammatory cytokines and promote the recovery of the intestinal barrier. We speculate that the mechanism of WHPs is closely related to the improvement of oxidative stress and the maintenance of the intestinal barrier. Therefore, WHPs have potential for application as nutritional agents by reducing the damage of radiotherapy of cancer patients [33].

## Figures and Tables

**Table 1:** Typical amino acid profile of whey hydrolysate peptides (WHPs).

Amino Acid	Content (g/100 g)
Glutamic Acid	14.4
Aspartic Acid	8.9
Leucine	8.6
Lysine	8.1
Threonine	5.8
Isoleucine	5.4
Proline	5.0
Valine	4.6
Alanine	4.3
Serine	4.2
Phenylalanine	2.6
Tyrosine	2.5
Arginine	2.3
Cystine/Cysteine	2.0
Methionine	1.8
Tryptophan	1.7
Glycine	1.5
Histidine	1.5
Hydroxyproline	<0.1

**Table 2:** Effect of WHPs on survival time (day) after irradiation.

Group	*n*	Median	Q25	Q75
Vehicle control	12	30 #	30	30
IR control	12	7 *	2	8
IR+Whey protein	12	8 *	2	14
IR+WHPs-L	12	11 *	7	11
IR+WHPs-M	12	11 *#	9	16
IR+WHPs-H	12	10 *	7	14

* The difference was statistically significant (*p* < 0.05) compared with the vehicle control group; # the difference was statistically significant (*p* < 0.05) compared with the IR control group. IR, irradiated mice; IR+WHPs-L, irradiated mice+low-dose whey hydrolysate peptides; IR+WHPs-M, irradiated mice+medium-dose whey hydrolysate peptides; IR+WHPs-H, irradiated mice+high-dose whey hydrolysate peptides.

**Table 3:** Effects of WHPs on body weight in irradiated mice (*n* = 12, $\bar{x}$ ± s).

Group	3rd Day Post-Irradiation		14th Day Post-Irradiation	
	Initial Weight (g)	Final Weight (g)	Initial Weight (g)	Final Weight (g)
Vehicle control	21.25 ± 0.75	21.90 ± 2.02	21.18 ± 0.98	20.60 ± 0.91 #
IR control	21.26 ± 1.12	20.68 ± 1.24	20.91 ± 2.31	18.59 ± 1.75 *
IR+Whey protein	21.21 ± 1.12	20.70 ± 1.91	20.98 ± 2.31	18.72 ± 1.09
IR+WHPs-L	21.23 ± 0.83	20.73 ± 2.10	21.02 ± 2.06	18.93 ± 1.17
IR+WHPs-M	21.35 ± 0.61	21.42 ± 1.58	20.84 ± 2.33	19.73 ± 1.45 #
IR+WHPs-H	21.23 ± 1.01	21.72 ± 1.35	21.00 ± 1.56	19.16 ± 1.85

* The difference was statistically significant (*p* < 0.05) compared with the vehicle control group; # the difference was statistically significant (*p* < 0.05) compared with the IR control group. IR, irradiated mice; IR+WHPs-L, irradiated mice+low-dose whey hydrolysate peptides; IR+WHPs-M, irradiated mice+medium-dose whey hydrolysate peptides; IR+WHPs-H, irradiated mice+high-dose whey hydrolysate peptides.

**Table 4:** Effects of WHPs on organ index in BALB/c mice at day three after radiation (*n* = 12, $\bar{x}$ ± s).

Group	Spleen Index (mg/g)	Thymus Index (mg/g)	Liver Index (mg/g)
Vehicle control	4.05 ± 0.31 #	2.15 ± 0.61 #	43.27 ± 3.18
IR control	1.69 ± 0.11 *	0.61 ± 0.18 *	42.38 ± 2.69
IR+Whey protein	1.67 ± 0.18 *	0.74 ± 0.16 *	42.32 ± 3.78
IR+WHPs-L	1.77 ± 0.35 *	0.78 ± 0.27 *	44.10 ± 9.51
IR+WHPs-M	1.62 ± 0.14 *	0.85 ± 0.15 *	41.04 ± 2.16
IR+WHPs-H	1.62 ± 0.19 *	0.72 ± 0.17 *	40.86 ± 3.78

* The difference was statistically significant (*p* < 0.05) compared with the vehicle control group; # the difference was statistically significant (*p* < 0.05) compared with the IR control group. IR, irradiated mice; IR+WHPs-L, irradiated mice+low-dose whey hydrolysate peptides; IR+WHPs-M, irradiated mice+medium-dose whey hydrolysate peptides; IR+WHPs-H, irradiated mice+high-dose whey hydrolysate peptides.

**Table 5:** Effects of WHPs on organ index in irradiated mice at day 14 after radiation (*n* = 12, $\bar{x}$ ± s).

Group	Spleen Index (mg/g)	Thymus Index (mg/g)	Liver Index (mg/g)
Vehicle control	4.31 ± 0.50	2.05 ± 0.33	45.68 ± 4.81
IR control	4.94 ± 1.09	1.96 ± 0.39	51.73 ± 9.46
IR+Whey protein	5.26 ± 0.91 *	2.21 ± 0.59	50.36 ± 6.73
IR+WHPs-L	4.68 ± 1.62	2.21 ± 0.84	43.02 ± 14.50 #
IR+WHPs-M	4.64 ± 0.87	2.06 ± 0.49	45.46 ± 6.93
IR+WHPs-H	4.53 ± 1.10	2.15 ± 0.56	44.96 ± 5.36

* The difference was statistically significant (*p* < 0.05) compared with the vehicle control group; # the difference was statistically significant (*p* < 0.05) compared with the IR control group. IR, irradiated mice; IR+WHPs-L, irradiated mice+low-dose whey hydrolysate peptides; IR+WHPs-M, irradiated mice+medium-dose whey hydrolysate peptides; IR+WHPs-H, irradiated mice+high-dose whey hydrolysate peptides.

**Table 6:** Effect of WHPs on white blood cell (WBC) count (*n* = 12, $\bar{x}$ ± s).

Group	Number of WBCs at 3rd Day Post-Irradiation (10^9^/mL)	Number of WBCs at 14th Day Post-Irradiation (10^9^/mL)
Vehicle control	11.5 ± 3.2 #	10.5 ± 1.2 #
IR control	1.8 ± 0.6 *	8.4 ± 2.1 *
IR+Whey protein	2.2 ± 0.6 *	9.1 ± 1.9
IR+WHPs-L	2.2 ± 0.6 *	8.9 ± 1.3
IR+WHPs-M	2.3 ± 0.8 *	9.1 ± 1.7
IR+WHPs-H	2.0 ± 0.0 *	10.2 ± 2.3 #

* The difference was statistically significant (*p* < 0.05) compared with the vehicle control group; # the difference was statistically significant (*p* < 0.05) compared with the IR control group. IR, irradiated mice; IR+WHPs-L, irradiated mice+low-dose whey hydrolysate peptides; IR+WHPs-M, irradiated mice+medium-dose whey hydrolysate peptides; IR+WHPs-H, irradiated mice+high-dose whey hydrolysate peptides.

**Table 7:** Effect of WHPs on antioxidant defense systems at day three after radiation (*n* = 12, $\bar{x}$ ± s).

Group	Liver SOD (ng/g)	Serum SOD (ng/mL)	Liver GSH-PX (ng/g)	Serum GSH-PX (ng/mL)	Liver MDA (nmol/g)	Serum MDA (nmol/mL)
Vehicle control	76.18 ± 12.74 #	9.00 ± 1.33 #	1403.24 ± 226.73 #	165.04 ± 20.93 #	80.63 ± 18.77 #	10.30 ± 1.83 #
IR control	45.95 ± 11.78 *	4.94 ± 0.46 *	732.13 ± 206.88 *	83.81 ± 14.01 *	137.37 ± 19.42 *	15.72 ± 2.43 *
IR+Whey protein	67.77 ± 12.29 #	6.60 ± 1.06 *#	1155.86 ± 255.98 *#	125.71 ± 25.19 *#	115.08 ± 19.92 *#	11.90 ± 0.91 #
IR+WHPs-L	60.37 ± 10.43 *#	8.10 ± 1.26 #	1268.84 ± 234.70 #	132.78 ± 23.91 *#	86.68 ± 21.02 #	13.70 ± 0.90 *#
IR+WHPs-M	54.05 ± 10.80 *	7.26 ± 0.81 *#	1045.10 ± 81.61 *#	115.8 ± 22.75 *#	104.01 ± 20.10 *#	13.66 ± 2.39 *#
IR+WHPs-H	53.26 ± 7.48 *	6.24 ± 1.36 *#	909.92 ± 86.49 *	114.33 ± 18.3 *#	105.84 ± 17.11 *#	13.75 ± 1.73 *

* The difference was statistically significant (*p* < 0.05) compared with the vehicle control group; #the difference was statistically significant (*p* < 0.05) compared with the IR control group. IR, irradiated mice; IR+WHPs-L, irradiated mice+low-dose whey hydrolysate peptides; IR+WHPs-M, irradiated mice+medium-dose whey hydrolysate peptides; IR+WHPs-H, irradiated mice+high-dose whey hydrolysate peptides.

**Table 8:** Effect of WHPs on antioxidant defense systems at day 14 after radiation (*n* = 12, $\bar{x}$ ± s).

Group	Liver SOD (ng/g)	Serum SOD (ng/mL)	Liver GSH-PX (ng/g)	Serum GSH-PX (ng/mL)	Liver MDA (nmol/g)	Serum MDA (nmol/mL)
Vehicle control	72.75 ± 16.01 #	8.07 ± 1.11 #	1351.07 ± 201.14 #	155.12 ± 13.32 #	97.39 ± 11.80 #	10.48 ± 2.74 #
IR control	39.18 ± 12.17 *	4.74 ± 1.06 *	558.30 ± 209.47 *	90.73 ± 16.65 *	152.20 ± 15.03 *	16.81 ± 2.51 *
IR+Whey protein	63.24 ± 5.50 #	7.57 ± 1.66 #	1067.09 ± 130.32 *#	133.32 ± 7.11 *#	104.52 ± 17.50 #	11.84 ± 2.03 #
IR+WHPs-L	73.07 ± 12.91 #	8.94 ± 1.11 #	1227.39 ± 174.08 #	150.04 ± 21.71 #	92.22 ± 14.96 #	12.01 ± 1.71 #
IR+WHPs-M	63.71 ± 10.46 #	7.24 ± 1.20 #	1166.78 ± 219.12 #	130.57 ± 26.04 *#	94.53 ± 22.25 #	13.21 ± 1.92 *#
IR+WHPs-H	56.39 ±12.65 *#	6.69 ± 1.28 *#	992.34 ± 193.28 *#	122.71 ± 16.75 *#	116.90 ± 15.73 *#	12.01 ± 1.50 #

* The difference was statistically significant (*p* < 0.05) compared with the vehicle control group; #the difference was statistically significant (*p* < 0.05) compared with the IR control group. IR, irradiated mice; IR+WHPs-L, irradiated mice+low-dose whey hydrolysate peptides; IR+WHPs-M, irradiated mice+medium-dose whey hydrolysate peptides; IR+WHPs-H, irradiated mice+high-dose whey hydrolysate peptides.

**Table 9:** Effect of WHPs on villus height and crypt depth in mice (μm, *n* = 8, $\bar{x}$ ± s).

Group	Villus Height (3 days)	Villus Height (14 days)	Crypt Depth (3 days)	Crypt Depth (14 days)
Vehicle control	311.5 ± 30.8	377.6 ± 43.6	81.8 ± 10.4	71.0 ± 3.0 #
IR control	281.7 ± 22.2	336.7 ± 19.3	87.6 ± 10.8	90.1 ± 15.0 *
IR+Whey protein	330.5 ± 29.9 #	381.6 ± 43.1	85.8 ± 10.8	81.1 ± 8.4
IR+WHPs-L	303.2 ± 31.0	350.4 ± 51.2	89.9 ± 8.6	88.5 ± 12.1 *
IR+WHPs-M	315.2 ± 41.4	369.1 ± 31.0	92.4 ± 12.2	84.3 ± 15.8
IR+WHPs-H	313.9 ± 39.7 #	364.4 ± 52.5	77.7 ± 15.0 #	92.0 ± 13.8 *

* The difference was statistically significant (*p* < 0.05) compared with the vehicle control group; # the difference was statistically significant (*p* < 0.05) compared with the IR control group. IR, irradiated mice; IR+WHPs-L, irradiated mice+low-dose whey hydrolysate peptides; IR+WHPs-M, irradiated mice+medium-dose whey hydrolysate peptides; IR+WHPs-H, irradiated mice+high-dose whey hydrolysate peptides.

**Table 10:** Effect of WHPs on the expression of occludin and ZO-1 proteins (AOD, *n* = 12, $\bar{x}$ ± s).

Group	Occludin (3 days)	ZO-1 (3 days)	Occludin (14 days)	ZO-1 (14 days)
Vehicle control	0.175 ± 0.016 #	0.165 ± 0.010 #	0.181 ± 0.011	0.164 ± 0.010
IR control	0.156 ± 0.010 *	0.152 ± 0.008 *	0.170 ± 0.007	0.155 ± 0.011
IR+Whey protein	0.162 ± 0.008	0.149 ± 0.016 *	0.172 ± 0.022	0.155 ± 0.013
IR+WHPs-L	0.163 ± 0.013	0.164 ± 0.009	0.186 ± 0.027	0.163 ± 0.013
IR+WHPs-M	0.168 ± 0.024	0.168 ± 0.020 #	0.184 ± 0.042	0.160 ± 0.014
IR+WHPs-H	0.165 ± 0.011	0.162 ± 0.007	0.178 ± 0.020	0.161 ± 0.021

* The difference was statistically significant (*p* < 0.05) compared with the vehicle control group; # the difference was statistically significant (*p* < 0.05) compared with the IR control group. IR, irradiated mice; IR+WHPs-L, irradiated mice+low-dose whey hydrolysate peptides; IR+WHPs-M, irradiated mice+medium-dose whey hydrolysate peptides; IR+WHPs-H, irradiated mice+high-dose whey hydrolysate peptides.

**Table 11:** Effect of WHPs on intestinal permeability in irradiated mice on third day (*n* = 12, $\bar{x}$ ± s).

Group	D-Lactate (μmol/L)	DAO (pg/mL)	LPS (EU/L)
Vehicle control	31.58 ± 5.96	757.54 ± 265.71	14.52 ± 2.46
IR control	49.36 ± 4.27	1398.84 ± 166.12	23.12 ± 2.14
IR+Whey protein	39.02 ± 6.53	1069.59 ± 187.01	19.97 ± 3.11
IR+WHPs-L	40.53 ± 4.49	969.10 ± 185.02	17.90 ± 2.80
IR+WHPs-M	40.04 ± 4.18	1068.36 ± 167.25	20.46 ± 2.69
IR+WHPs-H	42.54 ± 5.20	1123.73 ± 238.61	19.18 ± 2.63

IR, irradiated mice; IR+WHPs-L, irradiated mice+low-dose whey hydrolysate peptides; IR+WHPs-M, irradiated mice+medium-dose whey hydrolysate peptides; IR+WHPs-H, irradiated mice+high-dose whey hydrolysate peptides; DAO, Diamine Oxidase; LPS, Lipopolysaccharide.

**Table 12:** Effect of WHPs on intestinal permeability in irradiated mice on 14th day (*n* = 12, $\bar{x}$ ± s).

Group	D-Lactate (μmol/L)	DAO (pg/mL)	LPS (EU/L)
Vehicle control	33.58 ± 4.01	796.50 ± 206.96	14.82 ± 3.12
IR control	50.29 ± 6.06	1490.52 ± 237.45	23.49 ± 1.80
IR+Whey protein	36.59 ± 4.60	767.24 ± 175.31	17.93 ± 2.32
IR+WHPs-L	35.23 ± 6.23	924.16 ± 233.64	18.56 ± 1.88
IR+WHPs-M	38.01 ± 5.36	976.59 ± 197.92	18.51 ± 2.68
IR+WHPs-H	38.93 ± 6.06	965.65 ± 101.67	19.44 ± 1.82

IR, irradiated mice; IR+WHPs-L, irradiated mice+low-dose whey hydrolysate peptides; IR+WHPs-M, irradiated mice+medium-dose whey hydrolysate peptides; IR+WHPs-H, irradiated mice+high-dose whey hydrolysate peptides; DAO, Diamine Oxidase; LPS, Lipopolysaccharide.

## Data Availability

The data presented in this study are available on request from the corresponding author. The data are not publicly available due to privacy.

## Supplementary Materials

The following are available online at https://www.mdpi.com/2072-6643/13/3/816/s1.
