# Supplementary material for: Radioprotective Effect of Whey Hydrolysate Peptides against γ-Radiation-Induced Oxidative Stress in BALB/c Mice
Source: Nutrients. 2021 Mar 2;13(3):816. doi: 10.3390/nu13030816 (PMC7999902; doi:10.3390/nu13030816)
Supplement: Supplementary file 1 [file nutrients-13-00816-s001.pdf]

Supplementary File 1.

Table S1. Daily protein intake in each group per mice (g).

Group	The amount of protein supplemented	During pre-feeding period		During the 1st week of irradiation		During the 2nd week of irradiation	
		Protein provided in feed	Total protein intake	Protein provided in feed	Total protein intake	Protein provided in feed	Total protein intake
Vehicle control	-	2.562	2.562	2.712	2.712	2.022	2.022
IR control	-	2.075	2.075	2.671	2.671	0.675	0.675
IR+Whey	0.036	2.148	2.184	2.710	2.746	1.592	1.628
IR+WHPs-L	0.007	2.091	2.098	2.683	2.691	1.371	1.378
IR+WHPs-M	0.036	2.106	2.142	2.679	2.715	1.628	1.664
IR+WHPs-H	0.072	2.096	2.168	2.578	2.650	1.267	1.339

The protein content in the feed is estimated based on the actual food intake of the mice.
